# Body size in early life and risk of lymphoid malignancies and histological subtypes in adulthood

**DOI:** 10.1002/ijc.30044

**Published:** 2016-04-15

**Authors:** TienYu Owen Yang, Benjamin J. Cairns, Mary E. Kroll, Gillian K. Reeves, Jane Green, Valerie Beral, on behalf of the Million Women Study Collaborators

**Affiliations:** ^1^ Nuffield Department of Population HealthUniversity of OxfordOxfordOX3 7LFUnited Kingdom

**Keywords:** body mass index, obesity, childhood obesity, lymphoma, myeloma, lymphoid malignancies

## Abstract

Risk of adult lymphoid malignancy is associated with recent adiposity. Some have reported apparent associations with adiposity in childhood or early adulthood, but whether these associations are independent of recent adiposity is unknown. Birth weight, body size at age 10 years, clothes size at age 20 years, and recent body mass index (BMI) were recorded in 745,273 UK women, mean age 60.1 (SD 4.9) at baseline, without prior cancer. They were followed for 11 years, during which time 5,765 lymphoid malignancies occurred. Using Cox regression, a higher risk of lymphoid malignancy was strongly associated with higher recent BMI (RR=1.33, 95%CI 1.17‐1.51, for BMI 35+ vs <22.5 kg/m^2^), and this association remained essentially unchanged after adjustment for birth weight and body size at 10. Higher lymphoid malignancy risk was also associated with large size at birth, at age 10, and at age 20 years, but after adjustment for recent BMI, the significance of the associations with large size at birth and at age 10 years was sufficiently reduced that residual confounding by adult BMI could not be excluded; a weak association with large size at 20 years remained (adjusted RR =1.17, 95%CI 1.10–1.24 for large size at age 20 *vs*. medium or small size). We found no strong evidence of histological specificity in any of these associations. In conclusion, our findings suggest a possible role of adiposity throughout adulthood in the risk of lymphoid malignancy, but the independent contribution of body size at birth and during childhood appears to be small.

## Abstract 2

**What's new?**

Previous research has found a link between obesity and adult lymphoid malignancy risk. In this paper, the researchers report on the relative importance of body size not only at the time of diagnosis, but also at birth, childhood, and age 20. They collected data from more than 750,000 women followed up for a period of 11 years. They showed that BMI at the time of diagnosis was the strongest determinant of risk. Being overweight at age 20 also conferred a small additional risk of lymphoid malignancy. There was comparably little evidence to support an additional role of body size at birth or childhood.

**Abbreviations**
BMI: body mass index
CI: confidence interval
CLL/SLL: chronic lymphocytic leukaemia/small lymphocytic lymphoma
RR: relative risk

Prospective studies have generally shown that risk of lymphoid malignancy in adults increases with increasing body mass index, when body mass has been recorded relatively recently, in the decade or so before cancer diagnosis.^1^, ^2^ Some have reported on the association of these malignancies with adiposity in childhood and in early adulthood.^3^, ^4^, ^5^, ^6^, ^7^, ^8^, ^9^, ^10^ Since adiposity in early life is associated with adiposity in adulthood, the reported association between adiposity in early life and risk of lymphoid malignancies in adulthood could be explained by adiposity in adulthood. Previous studies have not been large enough to examine in detail whether the associations of lymphoid malignancies with body size in earlier life can be explained by adiposity in later life.

These associations may also vary by subtype of lymphoid malignancies. A large number of cases are required for prospective investigation of risk by subtype. The classification of haematological cancer might be affected by conversion of ICD‐O‐2 to ICD‐O‐3 codes at the start of the 21st century,^11^ or by other changes in cancer diagnosis or registration practice throughout time.^12^ The largest prospective report so far, with 2,074 cases in 1992–2007, investigated the association between body size in early adulthood and risk of lymphoid malignancies by histological subtypes.^10^ They reported a weak association with B‐cell lymphomas, but the confidence intervals were wide for more refined subtypes due to small numbers, and the potential bias due to change of disease classification was not addressed. Here we report the association between risk of lymphoid malignancies and indicators of body size at birth, in childhood, in early adulthood, and in later life, in a large prospective cohort of women with 5,765 cases of lymphoid malignancies, all of which were diagnosed after major revisions to the histological classifications and UK cancer registration systems in 2001.^11^, ^12^ We investigated the extent to which any associations with adiposity in early life are independent of adiposity in later life, and examined these associations by major histological subtypes.

## Methods

The details of the Million Women Study have been described elsewhere,^13^ and study questionnaires can be viewed on the study website (www.millionwomenstudy.org). Briefly, 1.3 million UK women were recruited through the national breast screening programme in 1996–2001. At recruitment they completed a postal questionnaire and provided information on various personal, health and lifestyle factors. A repeat questionnaire was sent approximately three years after recruitment, in which the participants provided updated and additional information, including birth weight, relative body size at age 10 years, clothes size at age 20 years, and current weight. This questionnaire forms the baseline for our analyses. All women were followed for cancer registration, emigration, and death, by linkage to National Health Service Central Registers. The study was approved by the Multi‐Centre Research Ethics Committee for Anglia and Oxford.

### Lymphoid malignancies

Through linkage to cancer registration, we identified all lymphoid malignancies in the cohort that occurred from 2001, when the current registration and classification system was introduced.^11^, ^12^ Lymphoid malignancies were coded using the International Classification of Disease, 10th revision (ICD‐10) with morphology codes according to ICD‐O‐3 from 2001, according to which cases were classified into Hodgkin lymphoma (9,650–9,667); mature B cell including diffuse large B‐cell (9,678–9,684), follicular (9,690–9,698), plasma cell neoplasms (9,731–9,734), chronic lymphocytic leukaemia/small lymphocytic lymphoma (*i.e.,* CLL/SLL: 9,670, 9,823) and other mature B cell (9,671, 9,673, 9,687, 9,689, 9,699, 9,760–9,762, 9,764, 9,826, 9,833, 9,940); mature T cell (9,700–9,719, 9,827, 9,831, 9,834, 9,948); Others (9,727–9,729, 9,835–9,837).

We focused on the relative risks for all lymphoid malignancies combined and for four common subtypes (>500 cases): diffuse large B‐cell, follicular, plasma cell, and CLL/SLL. Further updates to the 2001 WHO classification were published in 2008, primarily to subclassify the broad subtypes. The definition of broad subtypes remained largely unchanged, except for more refined criteria for CLL to take into account immunophenotypic results of monoclonal B cells in peripheral blood.^14^

### Early life and recent body size variables

Information was collected on birth weight, relative body size at age 10 years (plumper, average and thinner compared to peers), clothes size at age 20 years (the smallest size reported among 8 or less, 10, 12, 14, 16 and 18+) and body mass index (BMI) calculated from height reported at recruitment and weight reported at baseline (referred to as recent BMI hereafter), as described elsewhere.^15^, ^16^ In a validation study, self‐reported birth weight, body size at age 10 years and clothes size at age 20 years in Million Women Study participants were strongly correlated with birth weight and body mass indices assessed prospectively at relevant ages (10 and 21 years) among a subgroup of women (*n* = 541) who were also participants in the National Survey of Health and Development (NSHD), a birth cohort born in 1946.^17^ In another validation study where height and weight were measured in approximately 4,000 women in the Million Women Study, the correlation between BMI calculated from self‐reported current height and weight at recruitment and from height and weight measured about a decade later was 0.85.^18^

### Analysis

For all analyses, women were followed from either the time when they reported their body sizes (mean age 59.5 years, SD 4.9) or January 1, 2001, whichever was later, to any cancer, death, emigration, loss to follow‐up or December 31, 2013. Women with prior cancer before baseline were excluded. We used Cox regression to estimate hazard ratios, described here as relative risks, of cancer by categories of birth weight, relative body size at age 10 years, clothes size at age 20 years, and recent BMI, using age as the underlying time variable. Body size at 20 years was classified according to clothes size into categories: small (size <12), medium (12–14) and large (16 or larger). We also estimated and plotted the risk by cross‐classification of both early and recent body size variables. The category corresponding to the smallest body size was used as reference group in most analyses, but group‐specific confidence intervals (gs‐CIs) were calculated using Plummer's method^19^ and presented in Tables. This approach allows confidence intervals to be estimated for all categories, including the reference group, so that the significance of differences between any two categories can be inferred even if neither is the reference group. Conventional confidence intervals (CIs) were still described in texts or tables wherever comparisons were made to the reference group. Heterogeneity across subgroups, defined by body size categories, or across histological subtypes was examined using chi‐squared tests.

All analyses, unless otherwise stated, were stratified by year of birth (before 1940, 1940–1945, 1946‐), 10 regions of residence in Scotland and England, and socioeconomic status (Townsend social deprivation index,^20^ in quintiles), and were adjusted for adult height (<155, 155–159, 160–164, 165–169, 170–174, >=175 cm), smoking (never, past smoker who stopped <10 years or >=10 years ago, current smokers who smoked <15, 15–24 and >=25 cigarettes per day), strenuous exercise (never, once per week, more than once per week), alcohol consumption (0, 1–4, 5–14, 15–29, 30+ g/day)^21^ and BMI (<22.5, 22.5–24.9, 25.0–29.9, 30.0–34.9, 35+ kg/m^2^) at baseline. Women with missing values for any of the adjustment variables (<3% for each) were assigned to separate categories for that variable. All analyses were performed using Stata 14.1 (StataCorp, College Station, Texas). All *p*‐values are two‐sided.

## Results

All women who reported their relative body size at age 10 years, clothes size at age 20 years, and recent height and weight were included in the main analyses (*n* = 745,273); among these, birth weight was also reported by 428,485 women. Birth weight, relative size at age 10 or 20 years, and recent BMI (at mean age 59.5 [SD 4.9] years) were each associated with other characteristics of the women at birth, in childhood, in early adulthood and in later life (Table 1), but the patterns of these associations varied. For example, all four indicators of body size at different ages were associated with adult height, but not all in the same direction: women who reported larger birth weight and larger size at age 20 years tended to be taller, while those who reported being plumper than average at age 10 years or who had a larger recent BMI tended to be shorter. Table 2 shows indicators of body size at different ages (birth, age 10 years, age 20 years and around 60 years) were correlated with one another in a temporal manner, *that is,* correlations between body size variables were generally greater when the ages of indicators were closer. Although all correlations were highly significant (*p* < 0.001), the coefficients were not large (Spearman correlation coefficients < 0.4).

**Table 1:** Participant characteristics by measures of adiposity at birth, childhood, early adulthood and by recent body mass index and details of follow‐up

	Birth weight^a^			Relative size at age 10 years			Relative size at age 20 years			Recent body mass index (kg/m^2^)			All women
	<2.5 kg	2.5‐3.9 kg	>=4.0 kg	Thinner	Average	Plumper	Small	Medium	Large	<25.0	25.0‐29.9	30.0+	
Number of women (%)	139 097	149 853	139 535	234 328	397 982	112 963	224 602	286 770	233 901	339 645	279 653	125 975	745 273
Age at baseline (mean, SD)	59.8 (4.8)	59.6 (4.8)	59.8 (4.9)	60.3 (4.9)	60.2 (4.9)	59.3 (4.8)	59.7 (4.7)	60.2 (4.9)	60.4 (5.0)	59.9 (5.0)	60.3 (4.9)	60.0 (4.8)	60.1 (4.9)
Year of birth (mean, SD)	1942 (5)	1942 (5)	1942 (5)	1942 (5)	1942 (5)	1942 (5)	1942 (5)	1942 (5)	1941 (5)	1942 (5)	1941 (5)	1942 (5)	1942 (5)
Characteristics at baseline													
Adult height in cm (mean, SD)	160.7 (6.6)	162.6 (6.4)	163.9 (6.6)	162.6 (6.9)	162.1 (6.5)	161.9 (6.7)	159.9 (6.2)	162.4 (6.3)	164.3 (6.8)	163.3 (6.5)	161.6 (6.4)	160.6 (7.0)	162.2 (6.7)
Lowest socioeconomic quintile (%)	20	18	19	21	19	21	19	19	21	17	20	27	20
Current smoker (%)	12	11	12	11	12	15	13	12	12	14	11	9	12
Alcohol consumption in g/day (mean, SD)	6.3 (8.3)	7.0 (8.7)	7.0 (8.9)	6.4 (8.5)	6.6 (8.5)	6.5 (8.8)	7.1 (8.8)	6.8 (8.6)	5.6 (8.1)	7.3 (8.8)	6.3 (8.4)	4.6 (7.8)	6.5 (8.5)
Strenuous exercise less than 1 time per week (%)	57	55	56	59	56	59	56	56	60	52	59	69	58
Follow‐up (years)	10.9 (2.6)	10.9 (2.6)	10.8 (2.6)	10.8 (2.6)	10.9 (2.6)	10.9 (2.6)	10.8 (2.5)	10.9 (2.6)	10.8 (2.7)	10.9 (2.6)	10.9 (2.6)	10.7 (2.7)	10.9 (2.6)
Number of lymphoid malignancies	1024	1103	1153	1717	3149	899	1472	2122	2171	2470	2143	1152	5765

a Numbers of birth weight categories do not add up to total number because of missing values

**Table 2:** Spearman correlation coefficient between adiposity variables at birth, childhood, early adulthood and recent body mass index

	Birth weight	Relative size at age 10 years	Relative size at age 20 years	Recent body mass index
**Birth weight**	1.00			
**Relative size at age 10 years**	0.13	1.00		
**Relative size at age 20 years**	0.12	0.37	1.00	
**Recent body mass index**	0.02	0.15	0.32	1.00

Among women who reported all four variables (n=428,485). All correlations are highly significant (p<0.0001).

During an average of 10.9 years of follow‐up (8.1 million person‐years in total), 5,765 cases of lymphoid malignancies were registered, on average 6.7 (SD 3.4) years after baseline. The adjusted relative risks for lymphoid malignancies by body size variables are shown in Table 3 with group‐specific confidence intervals (gs‐CIs (capital C and I)), so that any two groups could be compared directly. After adjustment for age, year of birth, region, and socioeconomic status, large size at birth (*p* = 0.004), at age 10 years (*p* = 0.003), and at age 20 years (*p* < 0.001) were each associated with a higher risk of lymphoid malignancies. The associations were weakened after additional adjustment for height, smoking, exercise, and alcohol consumption (*p* = 0.05, *p* = 0.001, and *p* < 0.001, respectively). Associations were further weakened after additional adjustment for recent BMI. In particular, the significance of the associations with birth weight (*p* = 0.1) and with relative size at age 10 (*p* = 0.01) was substantially reduced.

**Table 3:** Body size at birth, childhood, early adulthood and risk of lymphoid malignancy

		Adjusted for age, year of birth, region, and socioeconomic status	Additionally adjusted for 4 factors,^a^ but NOT recent BMI	Additionally adjusted for 4 factors,^a^ AND recent BMI
	cases	(RR, 95% gs‐CI)	(RR, 95% gs‐CI)	(RR, 95% gs‐CI)
**Birth weight**				
<2.5 kg	424	1.00 (0.91–1.10)	1.00 (0.91–1.10)	1.00 (0.91–1.10)
2.5–2.9 kg	600	1.00 (0.92–1.08)	0.99 (0.92–1.08)	1.00 (0.92–1.08)
3.0–3.4 kg	1103	1.02 (0.96–1.08)	0.99 (0.94–1.05)	1.00 (0.94–1.06)
3.5–3.9 kg	616	1.06 (0.98–1.15)	1.03 (0.95–1.11)	1.02 (0.94–1.11)
4.0 kg or larger	537	1.22 (1.12–1.33)	1.16 (1.06–1.26)	1.15 (1.05–1.25)
		p=0.004	p=0.05	p=0.1
**Relative size at age 10 years**				
Thinner	1717	1.00 (0.95–1.05)	1.00 (0.95–1.05)	1.00 (0.95–1.05)
Average	3149	1.08 (1.04–1.12)	1.09 (1.05–1.13)	1.08 (1.04–1.12)
Plumper	899	1.14 (1.07–1.22)	1.15 (1.08–1.23)	1.10 (1.03–1.18)
		p=0.003	p=0.001	p=0.01
**Relative size at age 20 years**				
Small	1472	1.00 (0.95–1.05)	1.00 (0.95–1.05)	1.00 (0.95–1.06)
Medium	2122	1.08 (1.04–1.13)	1.05 (1.01–1.10)	1.04 (1.00–1.08)
Large	2171	1.33 (1.28–1.39)	1.25 (1.20–1.31)	1.19 (1.14–1.25)
		p<0.001	p<0.001	p<0.001

BMI: body mass index; gs‐CI: group–specific confidence interval; RR: relative risk

a Additionally adjusted for height, smoking, exercise, and alcohol consumption.

The overall association with birth weight became non‐significant after adjustment for recent BMI, and it appears that the original association was being largely driven by the largest birth weight group. The relative risk (RR) for birth weight >=4.0 kg *versus* <2.5 kg was 1.15 (95% conventional confidence interval [CI] 1.01–1.30). The association with relative size at age 10 years was driven by increases in risk in both average and plumper categories *versus* thinner categories (RR for average *vs*. thinner =1.08, 95% CI 1.02–1.15; RR for plumper *vs*. thinner = 1.10, 95% CI 1.02–1.20). The overall association with risk for clothes size at age 20 years was mainly due to the large size group (RR for large *vs*. small =1.19, 95% CI 1.11‐1.29). In further analyses we converted each index of body size into dichotomised groups: the relative risk (fully adjusted, including adjustment for recent BMI) for the highest birth weight category (>=4.0 kg) *versus* the rest (<4.0 kg) was 1.14 (95% CI 1.04–1.25); the relative risk for being average or plumper *versus* thinner at age 10 years was 1.09 (95% CI 1.03–1.15); the relative risk for have large *versus* medium or small size clothes at age 20 years was 1.17 (95% CI 1.10–1.24).

**Figure 1:**
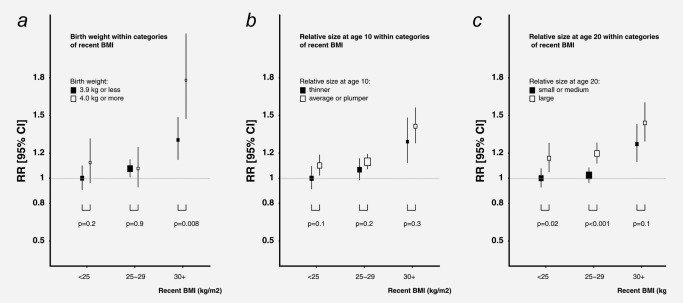
Relative risk of lymphoid malignancy by early body size variables within categories of recent body mass index (BMI). Body size variables were reported at about age 60 years, on average 7 years before lymphoid malignancy diagnosis. *P* values are for differences between pairs.

Using the dichotomised early body size categories for body sizes at birth, age 10 years, and age 20 years, we examined the risk of lymphoid malignancies by each index of body size within recent BMI (<25, 25‐29, 30+ kg/m^2^) as 2‐by‐3 categories to assess whether there were interaction effects*, for example,* whether the association with one measure of body size was dependent on recent BMI. The results are shown in Figure 1. We observed a significantly higher birth weight‐associated risk among women with high recent BMI (>=30 kg/m^2^) but not among other women (Fig. 1 *a*), with an overall heterogeneity of *p* = 0.04 across three recent BMI groups, but the confidence intervals were wide. The overall heterogeneity in the associations with relative size at age 10 years across subgroups of women by recent BMI was not significant (Fig. 1 *b*, *p* = 0.4). We did not find overall heterogeneity in the associations with clothes size at age 20 years, either (*p* = 0.2).

We also examined the relative risks of lymphoid malignancy using the dichotomised body size categories by major histological subgroups (Table 4) after multivariate adjustment including for recent BMI, where appropriate. For rarer subtypes the confidence intervals were wide due to small case numbers, and the large numbers of comparisons need to be taken into account in interpreting the results. Larger case numbers were seen in four main subtypes of mature‐B cell malignancy, including diffuse large B‐cell, follicular, plasma cell neoplasms, and CLL/SLL. Among the four subtypes, we did not see consistent associations with earlier body size variables, although there was significantly higher risk for plasma cell neoplasms among women reporting birth weight >=4.0 kg *versus* <4.0 kg (RR = 1.27, 95% CI 1.05–1.54), higher risk for diffuse large B‐cell neoplasms among women reporting plumper or average size at age 10 years compared to thinner size (RR = 1.16, 95% CI 1.02–1.33), and higher risk for both diffuse large B‐cell neoplasms and plasma cell neoplasms among women reporting large clothes size at 20 years compared to medium and small sizes (RR = 1.35, 95% CI 1.18‐1.54 for diffuse large B‐cell neoplasms and RR = 1.15, 95% CI 1.01–1.30). By contrast, having a recent BMI of 30 kg/m^2^ or more, compared to BMI < 30 kg/m^2^, was significantly associated with increased risk of diffuse large B‐cell, follicular and plasma cell subtypes of mature B‐cell malignancies (*p* < 0.05), but not with risk of CLL/SLL (Table 4, far right column).

**Table 4:** Body size at birth, childhood, and early adulthood and risk of lymphoid malignancies by histological subgroup

Relative risks^a^ and 95% confidence intervals associated with…						
				Relative size at age 10 years	relative size at age	
		Birth weight (>=4 kg vs. < 4kg)		(plumper vs. average/thinner compared to peers)	20 years (large vs.medium or small)	Recent BMI(>=30kg/m^2^ vs < 30kg/m^2^)
	cases	RR (95% CI)	cases	RR (95% CI)	RR (95% CI)	RR (95% CI)
**All lymphoid malignancies**	**3280**	**1.14 (1.04–1.25)**	**5765**	**1.09 (1.03–1.15)**	**1.17 (1.10–1.24)**	**1.27 (1.19–1.36)**
Hodgkin lymphoma (9650–9667)	116	1.07 (0.63–1.80)	193	0.88 (0.65–1.19)	1.03 (0.74–1.42)	1.62 (1.16–2.27)
Mature B cell	2629	1.14 (1.03–1.27)	4653	1.10 (1.03–1.17)	1.17 (1.10–1.25)	1.25 (1.16–1.35)
Diffuse large B–cell (9678–9684)	594	1.05 (0.84–1.32)	1081	1.16 (1.02–1.33)	1.35 (1.18–1.54)	1.37 (1.18–1.60)
Follicular (9690–9698)	487	1.26 (1.00–1.60)	839	1.11 (0.95–1.29)	1.12 (0.96–1.31)	1.29 (1.09–1.54)
Plasma cell neoplasms (9731–9734)	750	1.27 (1.05–1.54)	1325	1.03 (0.92–1.16)	1.15 (1.01–1.30)	1.23 (1.07–1.42)
CLL/SLL (9670, 9823)	520	0.88 (0.68–1.14)	886	1.00 (0.86–1.15)	1.03 (0.88–1.20)	1.12 (0.94–1.34)
Other mature B cell #	278	1.31 (0.96–1.78)	522	1.33 (1.09–1.62)	1.23 (1.01–1.50)	1.19 (0.95–1.49)
Mature T cell (9700–9719, 9827, 9831, 9834, 9948)	115	0.73 (0.41–1.32)	178	0.88 (0.64–1.20)	0.97 (0.69–1.36)	1.35 (0.94–1.96)
Others (9727–9729, 9835–9837)	420	1.28 (1.00–1.65)	741	1.14 (0.97–1.34)	1.22 (1.04–1.44)	1.31 (1.09–1.57)

BMI: body mass index; CLL/SLL: chronic lymphocytic leukaemia/small lymphocytic lymphoma; CI: confidence interval; MPD/MDD: myeloproliferative/myelodysplastic disorders; RR: relative risk

a Adjusted for age, year of birth, region, socioeconomic status, height, smoking amount and status, alcohol consumption, exercise, and recent BMI at an average age of 60 years, as appropriate

We have previously reported an increased risk of lymphoid malignancies in this cohort with increasing BMI recorded a decade or so before cancer diagnosis.^1^ In the analyses presented here, we found that the association with recent BMI, recorded at around age 60 years, an average of 7 years before diagnosis, remained after adjustment for body size in earlier life. As shown in Table 5, the increase in risk of lymphoid malignancy by baseline BMI remained highly significant and virtually unchanged after adjustment for birth weight and relative size at age 10 years. For example, the relative risk of lymphoid malignancies among women with recent BMI 35+ kg/m^2^ *versus* <22.5 kg/m^2^ was 1.33 (95% CI 1.17–1.51) before additional adjustment for birth weight or relative size at age 10 years, was 1.32 (95% CI 1.16–1.50) after additionally adjustment for birth weight, and 1.31 (95% CI 1.15–1.49) after additional adjustment for relative size at age 10 years. By contrast, the relative risk after adjustment for relative size at age 20 years was still significant but somewhat attenuated (RR = 1.20, 95%CI 1.05‐1.38, *p*<0.001).

**Table 5:** Risk of lymphoid malignancy by recent body mass index recorded at an average of 7 years before diagnosis: additional adjustment for body size at birth, childhood, and early adulthood

		Adjusted for age, year of birth, region, socioeconomic status and 4 other factors^a^	Additionally adjusted for birth weight	Additionally adjusted for relative size at age 10 years	Additionally adjusted for relative size at age 20 years
	cases	(RR, 95% gs‐CI)	(RR, 95% gs‐CI)	(RR, 95% gs‐CI)	(RR, 95% gs‐CI)
**Recent body mass index unit (kg/m^2^)**					
<22.5	1076	1.00 (0.94–1.06)	1.00 (0.94–1.06)	1.00 (0.94–1.06)	1.00 (0.94–1.07)
22.5–24.9	2700	1.01 (0.97–1.05)	1.01 (0.97–1.05)	1.01 (0.97–1.05)	0.99 (0.95–1.03)
25.0–29.9	1385	1.17 (1.11–1.23)	1.17 (1.11–1.23)	1.16 (1.10–1.22)	1.10 (1.05–1.16)
30.0–34.9	290	1.21 (1.07–1.35)	1.20 (1.07–1.35)	1.19 (1.06–1.33)	1.11 (0.99–1.24)
35.0–	314	1.33 (1.19–1.49)	1.32 (1.18–1.48)	1.31 (1.17–1.46)	1.20 (1.07–1.35)
		*p*<0.001	*p*<0.001	*p*<0.001	*p*=0.002

gs‐CI: group‐specific confidence interval; RR: relative risk; SE standard error

a †Additionally adjusted for height, smoking, exercise, and alcohol consumption.

## Discussion

In this large prospective cohort of women, there were apparent associations between large body size at birth, in childhood, and in early adulthood and risk of adult lymphoid malignancy. These early body size variables were correlated to varying degrees with recent body mass index, which was also significantly associated with risk of lymphoid malignancy. After allowing for recent adiposity, the association with body size in early adulthood (at around age 20) remained significant but weak. The significance of the associations with body size at birth and during childhood was, however, substantially reduced after adjustment for recent BMI.

Obesity has been linked to cancer through hormonal, immune, and inflammatory mechanisms.^22^, ^23^ An independent association between childhood obesity and adult lymphoid malignancies may suggest cumulative or latent risks through these mechanisms. Several previous studies have reported on the apparent risk of variously defined lymphoid malignancies associated with body size in childhood^3^, ^9^ and in early adulthood.^3^, ^4^, ^5^, ^6^, ^7^, ^8^, ^9^, ^10^ The largest prospective study among them, which included 2,074 incident cases, reported increased risks of non‐Hodgkin lymphoma associated both with current body size and with body size at age 18 years, but did not report whether the association at age 18 was independent of current adiposity.^10^ To minimise inconsistency in lymphoid malignancy diagnosis, we included only lymphoid malignancies diagnosed from January 1, 2001, and there were 5,765 incident lymphoid malignancies after this date. We had a sufficient number of cases to investigate associations after subdividing the data both by body size at younger ages and by recent BMI. It was also possible to examine findings by major histological subtype according to more consistent and recent guidelines of diagnostic criteria.^11^

The main limitation of this study is that we were unable to exclude the possibility of residual confounding by body size between the ages for which information was recorded. The increased risk of lymphoid malignancy associated with large body size at age 20 and with recent BMI cannot be interpreted as representing associations that are specific to body size at those particular ages, as we do not have information on body size during the intervening years. The smaller and statistically weaker associations with body size at birth and at age 10 years might be affected by measurement errors which could attenuate estimates and reduce power to detect an association, but might also be confounded by associations with body size in adult years that could not be taken account in analyses. There was no strong evidence for differences in results by histological subtypes among body size at birth, age 10, and age 20. The significantly higher risk associated with large birth weight (4.0+ *vs*. <4.0 kg) was consistent with our previous report on birth weight and non‐Hodgkin lymphoma in this cohort,^15^ but this association was largely confined to the subgroup of women with recent BMI 30+kg/m^2^, and could be due to chance.

Our findings were based on self‐reported body size variables. Our validation studies^17^, ^18^ have demonstrated that self‐reported categories reflected differences in body size at relevant ages, but possible confounding through unaccounted reporting errors cannot be excluded. Self‐reported body size at four different time periods were weakly or moderately correlated, this could be due to true variability of adiposity throughout life and/or measurement errors.

With sufficient cases diagnosed from 2001, presumably when most cases were defined by the updated diagnostic and registration system (except CLL), this report is more statistically powerful than previous studies for histology‐specific associations with adiposity at birth, in childhood,^3^, ^9^ and in early adulthood,^3^, ^5^, ^6^, ^7^, ^8^, ^10^, ^24^, ^25^, ^26^, ^27^, ^28^ especially the four most common mature B‐cell malignancies (*n* > 500 for diffuse large B cell, follicular, plasma cell, and CLL/SLL). Consistent with our previous report,^1^ we found that recent BMI was significantly associated with diffuse B cell, follicular, and plasma cell subtypes. There was no strong evidence suggesting risk of any subtype was in particular affected by adiposity in early life, despite some associations identified at relatively weak significance. Even with our criteria for cases diagnosed from 2001, misclassification is still possible and may potentially cause biases if it was associated with risk factors. Even with the largest case number so far, we were not able to investigate more refined categories, and collaborative efforts, such as the International Lymphoma Epidemiology Consortium (InterLymph),^29^ will be important in understanding histology‐specific associations.

In conclusion, in this large cohort of UK women, risk of lymphoid malignancies was strongly associated with high recent BMI, an average of 7 years before the lymphoid malignancies were diagnosed, and weakly associated with BMI in early adulthood, suggesting a possible role of adiposity throughout adult life. Large body sizes at birth and in childhood appeared to contribute relatively little to the risk of adult lymphoid malignancies.

## Authorships

All authors read and approved the submission of this manuscript.

All authors declared no conflict of interest.

TOY designed research, analysed data, and wrote the paper.

BJC designed research, critically reviewed and jointly wrote the paper.

MEK designed research and critically reviewed the paper.

GKR designed research, critically reviewed and jointly wrote the paper.

JG designed research, critically reviewed and jointly wrote the paper.

VB designed research, critically reviewed and jointly wrote the paper.
